# Overexpression of the Lung Cancer-Prognostic *miR-146b* MicroRNAs Has a Minimal and Negative Effect on the Malignant Phenotype of A549 Lung Cancer Cells

**DOI:** 10.1371/journal.pone.0022379

**Published:** 2011-07-18

**Authors:** Santosh Kumar Patnaik, Eric Kannisto, Reema Mallick, Sai Yendamuri

**Affiliations:** 1 Department of Thoracic Surgery, Roswell Park Cancer Institute, Buffalo, New York, United States of America; 2 Northeastern Ohio Universities College of Medicine, Rootstown, Ohio, United States of America; 3 Department of Surgery, University at Buffalo, Buffalo, New York, United States of America; Karolinska Institutet, Sweden

## Abstract

**Introduction:**

Expression levels of *miR-146b-5p* and *-3p* microRNAs in human non-small cell lung cancer (NSCLC) are associated with recurrence of the disease after surgery. To understand this, the effect of *miR-146b* overexpression was studied in A549 human lung cancer cells.

**Methods:**

A549 cells, engineered with lentiviruses to overexpress the human *pre-miR-146b* precursor microRNA, were examined for proliferation, colony formation on plastic surface and in soft agar, migration and invasiveness in cell culture and in vivo in mice, chemosensitivity to cisplatin and doxorubicin, and global gene expression. *miR-146b* expressions were assessed in microdissected stroma and epithelia of human NSCLC tumors. Association of *miR-146b-5p* and *-3p* expression in early stage NSCLC with recurrence was analyzed.

**Principal Findings:**

A549 *pre-miR-146b*-overexpressors had 3–8-fold higher levels of both *miR-146b* microRNAs than control cells. Overexpression did not alter cellular proliferation, chemosensitivity, migration, or invasiveness; affected only 0.3% of the mRNA transcriptome; and, reduced the ability to form colonies in vitro by 25%. In human NSCLC tumors, expression of both *miR-146b* microRNAs was 7–10-fold higher in stroma than in cancerous epithelia, and higher *miR-146b-5p* but lower *-3*p levels were predictive of recurrence.

**Conclusions:**

Only a minimal effect of *pre-miR-146b* overexpression on the malignant phenotype was seen in A549 cells. This could be because of opposing effects of *miR-146b-5p* and *-3p* overexpression as suggested by the conflicting recurrence-predictive values of the two microRNAs, or because *miR-146b* expression changes in non-cancerous stroma and not cancerous epithelia of tumors are responsible for the prognostic value of *miR-146b*.

## Introduction

MicroRNAs are ultrashort non-coding RNAs that regulate cellular processes by their sequence-specific influence on the stability and translation of multiple messenger RNAs (mRNAs). Studies over the past few years have shown the importance of these epigenetic regulatory molecules in cancer biology [1] as well as their utility as biomarkers of disease states [2]. We previously quantified the expression of microRNAs in 77 human stage I non-small cell lung cancer (NSCLC) tumors to identify microRNAs whose levels are associated with recurrence of the disease following surgical resection [3]. Expression of *miR-146b* microRNAs, *miR-146b-5p* and *miR-146b-3p*, in the tumors differed significantly between cases that had a recurrence and those that did not, with the mean *miR-146b-5p* level higher but the mean *miR-146b-3p* level lower in the former group. Furthermore, in internal cross-validations, *miR-146b-3p* was identified as a frequent constituent of six-microRNA support vector machines classifiers that could predict recurrence with a mean accuracy of 69%. Higher expression of *miR-146b-5p* in human tumors was also found to be associated with poor overall survival in stage I-III NSCLC in a study that focused on the squamous cell variety of the disease but did not examine *miR-146b-3p* [4]. Besides lung cancer, a higher level of *miR-146b-5p* in tumors is also associated with a more malignant disease in human papillary thyroid carcinoma [5].

In humans, both *miR-146b-5p* and *-3p* microRNAs are products of the same *MIR146B* gene that is located on chromosome 10 at position q24.32. Like most pairs of 5p and 3p microRNAs, the two *miR-146b* microRNAs are generated from the opposite strands (the 5′ and 3′ arms) of a double-stranded stem region of a precursor microRNA, *pre-miR-146b*, which in turn is derived from the primary *MIR146B* RNA transcript [6]. The expression of *miR-146b-5p* appears to be ubiquitous in human tissues, though higher levels are seen in lung, thymus and spleen [7]. Small RNA cloning and deep sequencing studies show that the amount of *miR-146b-3p* in cells is much less than that of *miR-146b-5p* [8], [9]. Such a difference is seen for many 5p and 3p microRNA pairs, which constitute a majority of microRNAs. This strand bias is believed to be a result of thermodynamic effects on microRNA processing dictated by the microRNA sequences [10], [11] as well as the levels of target mRNAs in the cellular environment [12], [13]. Even though one of the 5p and 3p microRNAs may be much less abundant than the other (and often referred to as the 'microRNA*' species), it can have significant influence on the cellular state [14], [15]. It should be noted that the nature of strand bias can vary spatiotemporally. For example, the *miR-202*/miR-202* expression ratio changes from ∼0.5 to ∼0.9 during female gonadogenesis in chicken embryos [16], and mouse *miR-194-5p* is present at a higher level than *miR-194-3p* in kidney and stomach, but at a lower level in lung and uterus [17].

A number of studies have examined the role of *miR-146b* microRNAs in cancer ceIl-lines. In a study of U373 human glioblastoma cells, invasiveness and migration was decreased by overexpression of the microRNAs and increased by their knockdown using antisense oligonucleotides, and the targeting of transcripts of the *MMP16* matrix metalloproteinase gene by *miR-146b-5p* was identified [18]. In vitro migration and invasion of another human glioblastoma cell-line, U87-MG, has also been shown to be reduced by *miR-146b-5p* overexpression [19]. The microRNA was shown to target transcripts of the *EGFR* gene encoding epidermal growth factor receptor. *EGFR*-targeting by *miR-146b-5p* has also been shown in MDA-MB-231 human breast cancer cells [20]. As in the glioblastoma studies, *pre-miR-146b* overexpression in the cells reduced thier migration and invasiveness [20]. Similar findings were reported in another study which also suggested a role for *miR-146b-5p* in negatively regulating the nuclear factor-kB (NF-kB) pathway [21]. Such functional studies have not been performed in lung cancer cells. To understand the association of the *miR-146b* microRNAs with lung cancer prognosis, we sought to study the effects of overexpressing the *pre-miR-146b* precursor microRNA on the malignant phenotype of the A549 lung adenocarcinoma cell-line.

## Materials and Methods

### Ethics statement

All research presented here was approved by the Institutional Review Board of the Roswell Park Cancer Institute (RPCI). All animal experiments were performed after approval by RPCI's Institute Animal Care and Use Committee under protocol number 1132M.

### Cell culture

A549 human lung adenocarcinoma and BEAS-2B normal human bronchial epithelial cell-lines were obtained from ATCC® (Manassas, VA). TLA-HEK293T™, a human embryonic kidney cell-line derived from the 293T cell-line, was obtained from Open Biosystems® (Huntsville, AL). A549 and TLA-HEK293T™ cells were cultured at 37°C in 90% humidity and 5% CO_2_ in Dulbecco's modified Eagle medium (+DMEM; Mediatech®, Manassas, VA) with 10% fetal bovine serum (PAA Laboratories®, Pasching, Austria) in a Steri-Cult™ incubator (Thermo Fisher Scientific®, Waltham, MA). LHC-9 medium (Invitrogen®, Carlsbad, CA) was used for BEAS-2B cells. Cell-lines generated in this study were cultured in the presence of 2 µg/ml puromycin (Roche®, Indianapolis, IN). Cells were harvested using 0.05% trypsin with 0.25 mM ethylene-diamine-tetraacetic acid (Invitrogen®). Cells were not cultured for more than six weeks, and concurrently-cultured and similarly-aged cells were used when comparing cell-lines. Maintenance cultures at semi-confluence were used to set up experiments. A Scepter™ electronic counter (Millipore®, Billerica, MA) or disposable hemacytometer slides (Incyto®, Dusseldorf, Germany) were used to measure cell-densities of re-suspended cells.

### Generation of stably-transduced cell-lines

Complementary, single-stranded DNA oligonucleotides with the human *pre-miR-146b* sequence (miRBase [22] accession number MI0003129) and restriction enzyme-site overhangs were obtained from Integrated DNA Technologies® (Coralville, IA). Oligonucleotides were also obtained for a variant sequence in which the segments for the *miR-146b-5p* and *-3p* microRNAs were swapped. After annealing to generate double-stranded DNA, the oligonucleotides were ligated to *Xho* I- and *Not* I (Invitrogen®)-digested pLemiR™ vector (Open Biosystems®). TOP10™ competent *E. coli* (Invitrogen®) were heat-transformed with the ligation products. Plasmid DNA was prepared from cultures of selected single clones using a kit supplied by Qiagen® (Valencia, CA). Identity of plasmids was confirmed by DNA sequencing at a core facility at the Roswell Park Cancer Institute in Buffalo, NY (RPCI). Engineered lentiviruses were prepared by transfecting plasmids into TLA-HEK293T™ cells using reagents and protocols provided with the Trans-Lentiviral GIPZ™ packaging system (Open Biosystems®). A549 cells were transduced with viruses for 48 hours and then subjected to selection against 2 µg/ml puromycin for about two weeks to generate cell-lines A549/vec, A549/146b, and A549/v146b, using viruses generated with an empty pLemiR™ vector, or with the vector bearing the natural or the variant *pre-miR-146b* sequence, respectively.

### Flow cytometry

Flow cytometry of freshly-harvested cells stained with 1 µg/ml of the *7*-amino-actinomycin D viability dye (Sigma®, St. Louis, MO) was performed on a FACSCalibur machine (BD Biosciences®, San Jose, CA). Forward- and side-scatter, and fluorescence in the FL2 and FL4 channels were recorded for 10,000 events, and FL2 fluorescence values were plotted after gating out dead cells with the CellQuest™ Pro software (version 5; BD Biosciences®).

### Cell proliferation assay

Cell proliferation assays were performed using the Cell Counting Kit-8 assay (CCK-8; Dojindo Molecular Technologies®, Rockville, MD) which uses the bioreduction of *2*-(*2*-methoxy-*4*-nitrophenyl)-*3*-(*4*-nitrophenyl)-*5*-(*2*,*4*-disulfophenyl)-2H tetrazolium sodium (WST-8) to orange-colored formazan to measure cell viability. Briefly, 100 µl of cells at 2-4x10^4^/ml cells were plated on 96-well culture plates. At various time-points over the next four days, the culture medium in quadruplicate wells was replaced with fresh medium containing 5% CCK-8 reagent, and after an hour, absorbance at 450 nm was measured on a Multiskan Plus (MTX Lab Systems®, Vienna, VA) plate reader. Absorbance values were corrected by subtracting those of control wells that did not have cells.

### Drug sensitivity assay

Cells were grown to 50%-60% confluence in 96-well culture plates in quintuplicate when the culture medium was replaced with that containing 0.5–2.0 µg/ml of either doxorubicin hydrochloride (Enzo Life Sciences®, Farmingdale, NY) or cisplatin (Calbiochem®, San Diego, CA) or neither. After two days of culture, viability of cells was measured as described for the cell proliferation assay.

### Colony formation assays

For adherent colony formation assays, 200 cells were plated per 35 mm-culture dish in triplicate and cultured with medium changes every 4–5 days. After 10–14 days, dishes were stained with 0.2% methylene blue in 40% methanol for 30 minutes. For colony formation in soft agar, a dish was first coated with 1.5 ml medium containing 0.7% agar (Sigma®) before cells, in 2 ml medium containing 0.35% agar, were added at 150 cells/dish. After polymerization of agarose in about 15 minutes, 2 ml of medium was added. Cells were cultured for 6–7 weeks with medium change every 4–5 days, and then stained with 2 mg/ml thiazolyl blue tetrazolium bromide (Sigma®) for 4–8 hours in the cell culture incubator. Stained dishes were scanned on a Perfection V700 (Epson®, Long Beach, CA), and a minimum area on images corresponding to 3778 or 199 µm^2^, respectively for the adherent and soft agar colony assays, was used for identifying colonies for quantification using ImageJ™ software (version 10.2; National Institute of Mental Health, Bethesda, MD).

### In vitro wound healing assay

Six-well culture plates were marked with a cross-hair pattern on the outside surface of their bottoms. Cells were then plated in triplicate at a high density to reach 100% confluence a day later, when an approximately 2 cm-long and 0.5 mm-wide wound was made by dragging a plastic 10 µl pipette-tip along a straight edge with moderate and consistent pressure to scratch the cell monolayer. The culture medium was then replaced. Cells were imaged immediately and after a day of culture at 50x magnification at the same locations along the scratches.

### Transwell™ migration and invasion assays

Twenty-four-well Transwell™ plates with a polycarbonate membrane and an 8 µm pore-size were obtained from Corning® (Corning, NY). Semi-confluent cells that had been starved for 16–20 hours by culture in serum-free +DMEM medium were harvested from 10 cm-culture dishes, washed twice with phosphate-buffered isotonic saline (PBS; 137 mM NaCl, 2.7 mM KCl, 10 mM Na_2_HPO_4_ and 1.76 mM KH_2_PO_4_ at pH 7.4), and re-suspended in serum-free +DMEM medium at 2–3×10^5^ cells/ml. Cells were plated in triplicate in 0.1 ml of +DMEM per Transwell™, with bottom wells containing 0.6 ml +DMEM medium with 15% fetal bovine serum. The same volume of cell suspensions were concurrently plated in triplicate in wells of 24-well culture plates for indirectly assessing cell density by measuring cell viability after 8–16 hours as described for cell proliferation assay. For invasion assays, the Transwell™ membranes were pre-coated with either Cultrex™ PathClear™ basement membrane extract prepared from murine Engelbreth-Holm-Swarm tumor cells, or collagen I from rat tails (Trevigen®, Gaithersburg, MD). Coating was performed by layering 50 µl of the proteins at 1.5 mg/ml and 50 µg/ml, respectively, in serum-free +DMEM medium and allowing the layers to dry under 90% humidity at 37°C for 1 hour and 16–24 hours, respectively. Dried layers were rinsed thrice with serum-free +DMEM medium before cells were plated. After 8 or 16–20 hours, respectively, for the migration or invasion assays, upper surfaces of the Transwell™ membranes were wiped with cotton swabs to remove non-migrated cells. In the migration assay, cells remaining in the membranes were fixed with 10% neutral buffered-formalin and stained overnight with 0.2% w/v crystal violet (Sigma®) in 2% ethanol. After thorough washing with water to remove any unincorporated dye, 300 µl of 10% acetic acid was added to each Transwell™ to extract bound dye by shaking the wells at 100 revolutions/minute for 20 minutes. Extracts were analyzed for absorbance at 595 nm using 10% acetic acid as reference. In the invasion assays, red fluorescence images of cells in the Transwell™ membranes were acquired at 50x magnification in three different field-views. The absorbance readings or the average number of cells per field was normalized using the cell viability measurements of the cells that had been concurrently plated in separate cultures.

### Pulmonary tumor formation assay

This work was approved by various institutional committees at RPCI. Cells grown to about 70% confluence were harvested by trypsinization for three minutes, washed with PBS, and re-suspended in ice-cold +DMEM at 5×10^6^ cells/ml. An expert technician used a 1 ml-syringe with a 27 G needle to inject 0.2 ml of cells in the tail vein of immunodeficient, female, 6–10 week-old mice of C.B-*Igh*-*1^b^*/lcrTac-*Prkdc^scid^*/Ros strain. Mice were monitored twice weekly for labored breathing as well as for general malaise and any visible appearance of a cancerous lesion or growth, and euthanized after 12 weeks even though none of the mice had any of these symptoms or signs. Lungs were removed, washed with PBS, fixed for a day with 10% neutral buffered-formalin, and weighed after dabbing off excess liquid with paper towels.

### Laser capture microdissection (LCM)

This work was approved by an institutional review board at RPCI, and performed under the supervision of a pathologist in the Department of Pathology of RPCI on tissues from 12 cases of stage I NSCLC that had been treated at the institute. Formalin-fixed, paraffin-embedded, 6–8 µm-thick tissue-sections with at least 70% tumor tissue as verified by histological examination by a pathologist were used. Tissue-sections were placed on glass slides coated with a polyethylene naphthalate membrane (Leica®, Wetzlar, Germany) and dried overnight at room temperature. After de-paraffinization with xylene followed by gradual rehydration overnight using graded alcohols, the slides were stained with hematoxylin and eosin, and air-dried overnight. LCM was performed on an LMD6000 system (Leica®) at 200x magnification with laser power, speed and specimen-balance settings of 80, 2 and 11, respectively. Tumor-containing regions on the sections were identified by the altered cellular morphology of cancerous epithelial cells. Stromal and cancerous epithelial components of such regions were microdissected and collected in separate 0.5 ml tubes. For each component, areas of 1–12×10^6^ µm^2^ (approximately 0.5–7×10^4^ cell-sections) were collected. The stromal-to-epithelial area ratio varied from about 0.6 to 1.

### Isolation of RNA

Silica columns, reagents and protocols provided with the Purelink™ RNA (Invitrogen®), or High Pure™ microRNA (Roche®) and FFPE RNA (Norgen Biotek®, Thorold, Canada) kits, respectively, were used to isolate total from 2–5×10^6^ cultured cells or from proteinase K-treated microdissected cells. Nucleic acid concentration in the preparations was estimated by absorbance spectrophotometry on Ultrospec II (LKB Biochrom®, Cambridge, UK) or NanoDrop™ 1000 (Thermo Fisher Scientific®) instruments. A fluorometric assay using Quant-iT™ Ribogreen (Invitrogen®) was used to quantify nucleic acids in the preparations from the microdissected cells. Total RNA from MCF-7 human breast cancer and ML-2 human acute myeloid leukemia cells were kindly provided by, respectively, the research groups of Drs. Gokul Das and Eunice Wang of RPCI.

### Semi-quantification of microRNAs by reverse transcription-PCR (RT-PCR)

Semi-quantification of human microRNAs *let-7a*, *miR-30a-5p*, *miR-146b-5p* and *miR-146b-3p*, and the small nucleolar RNA *RNU6B* was done with RT-PCR-based TaqMan™ MicroRNA Assays (Applied Biosystems®, Foster City, CA; assay IDs 377, 417, 1097, 2361 and 1093, respectively). Briefly, an RNA-specific oligonucleotide, and reagents provided with the TaqMan™ MicroRNA Reverse Transcription Kit (Applied Biosystems®) were used to reverse transcribe 10 ng total RNA from cells, or 5 µl of 0.5-2000 fM synthetic mature microRNAs *miR-146b-5p* and *-3p* with 5′ phosphate and 3′ hydroxyl terminii obtained from Invitrogen®. The cDNA was used as template in 40 cycle-PCR reactions on a 7900HT real-time PCR machine (Applied Biosystems®). For each reaction, the quantification cycle (C*_q_*) value, approximately inversely proportional to log_2_ value of the concentration of the analyte RNA, was obtained with SDS™ software (Applied Biosystems®; version 2.3). The average of C*_q_* values of triplicate PCR reactions was used for analysis. When needed, C*_q_* values for microRNAs were normalized by subtracting the value for the small nucleolar *RNU6B* RNA.

### Gene expression analysis using BeadChip™ technology

Total RNA, isolated from A549/vec and A549/146b cells in two experiments for biological duplication, was provided to the shared gene expression facility of RPCI for analyses. Absorbance spectrophotometry and electrophoresis on NanoDrop™ 1000 and Bioanalyzer™ 2100 (Agilent Technologies®, Santa Clara, CA), respectively, showed that the RNA preparations had 260 nm/280 nm absorbance ratios in the 1.9–2.0 range, 260 nm/230 nm absorbance ratios >1.8, and RNA integrity numbers >7. Illumina® TotalPrep RNA Amplification kit (Ambion®, Autin, TX) was used to convert 500 ng of RNA to cDNA, which was then transcribed in vitro to generate biotin-labeled RNA. Hybridization reagents were mixed with 750 ng of labeled RNA, and the mixture was hybridized overnight at 58°C to HumanRef-8 v3.0 Expression BeadChips™ array (Illumina®, San Diego, CA) that has probes to detect 24,526 annotated human transcripts. Following washing and staining with Cy3 dye-labeled streptavidin, BeadChips™ were imaged using the BeadArray Reader (Illumina®). Data was acquired with GenomeStudio™ software (version 2010.1; Illumina®), and then processed for background-correction, variance-stabilizing transformation, and then robust splines-normalization between arrays using the lumi Bioconductor package (version 1.14) for R language (version 2.11.1) with default settings. Raw and processed data can be found in the NCBI Gene Expression Omnibus repository with accession number GSE29496. Good quality of the data was confirmed by examine different aspects of data as recommended by Illumina®. Multi-testing with a paired t test with moderated t statistics and Benjamini-Hochberg correction for false discovery rate was done on the processed data using the limma Bioconductor package (version 3.4.5).

### Other

An EOS 450D digital camera (Canon®, Lake Success, NY) and an Axio™ Observer microscope (Zeiss®, Maple Grove, MN) were used for microphotography. Unless specified otherwise, chemicals were procured from Sigma® or Thermo Fisher Scientific®. RNA secondary structure prediction using mfold [23] with default settings was performed online at http://mfold.rna.albany.edu. Statistical analyses and graph plotting were done with Prism™ software (version 5.0c; GraphPad Software®, La Jolla, CA). All t tests were two-tailed, assumed equal variances, and used 0.05 as the cut-off for deciding significance.

## Results

### Generation of A549 cell-lines overexpressing *miR-146b* microRNAs

**Figure 1:**
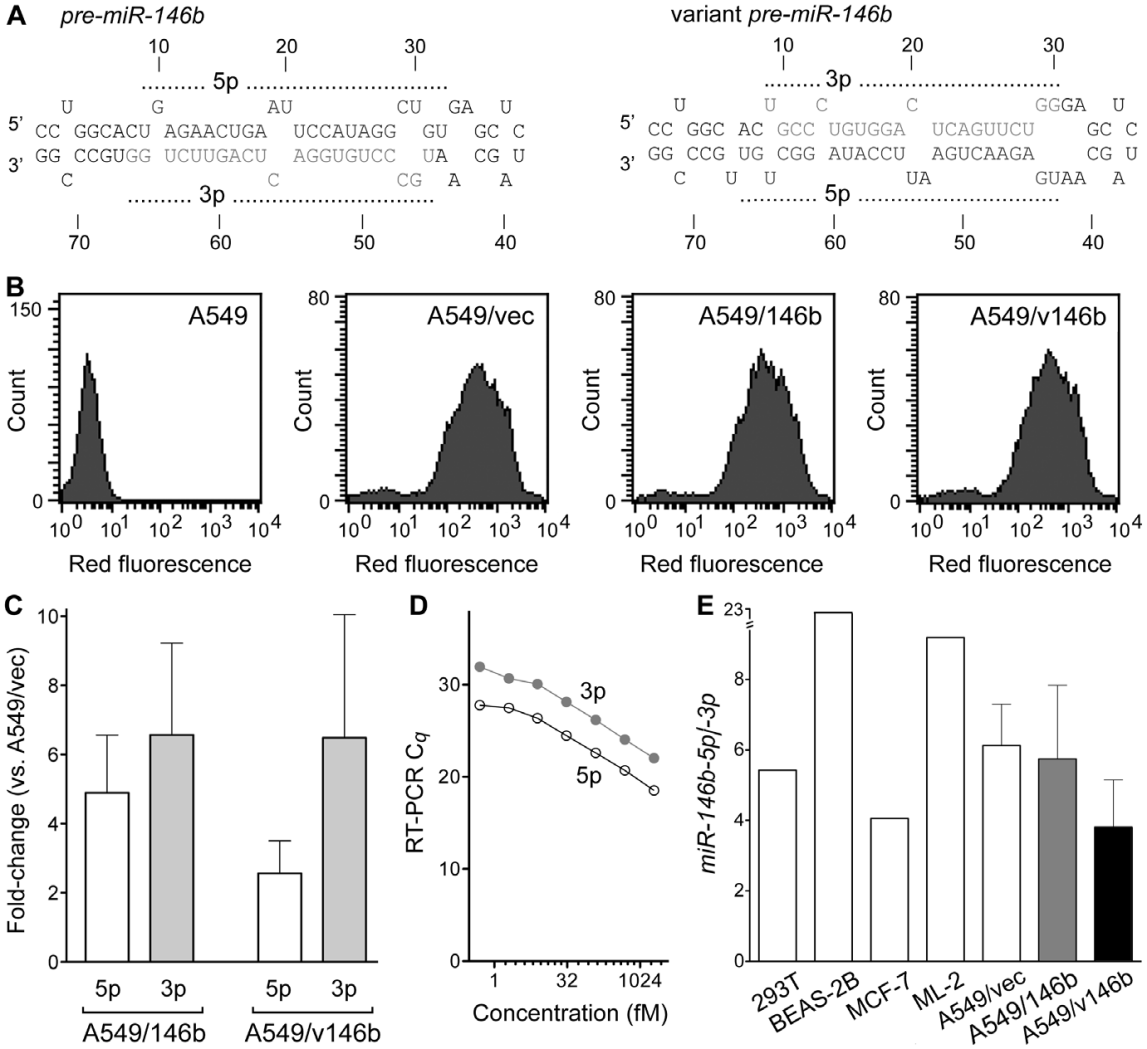
Generation of A549-derived cell-lines. *A*. Most stable mfold-predicted secondary structures of human *pre-miR-146b* and a variant pre-microRNA examined in this study. The 5′ and 3′ ends, nucleotide positions, and segments corresponding to mature *miR-146b-5p* and *-3p* sequences (*5p* and *3p*) are indicated. *B*. Histograms for red fluorescence quantified by flow cytometry of A549 and A549-derived cell-lines stably transduced with lentiviruses bearing constructs engineered for expression of human *pre-miR-146b* (*A549/146b*), or its variant shown in *A* (*A549/v146b*), or neither (*A549/vec*). *C*. Comparison of *miR-146b-5p* and *-3p* levels (*5p* and *3p*), normalized to that of the *RNU6B* small nucleolar RNA, in the A549-derived cell-lines as assessed using reverse transcription followed by PCR (RT-PCR). Means and their standard errors for measurements from three different experiments are shown. *D*. Standard curves showing the relationship between quantification cycle (*C_q_*) value and concentration of RNA in *miR-146b-5p* and *-3p* (*5p* and *3p*) RT-PCR assays of synthetic *miR-146b-5p* and *-3p* RNA, respectively. A log_2_ scale is used for the X axis. *E*. Semi-quantification of the relative amounts of *miR-146b-5p* and *-3p* in RNA of 293T, BEAS-2B, MCF-7, and ML-2 (means, single experiments), and the three A549-derived cell-lines, (means and their standard errors for measurements from three different experiments) are shown.

To study the effect of *miR-146b* overexpression in lung cancer cells, human A549 lung adenocarcinoma cells were engineered to overexpress the human *pre-miR-146b* precursor microRNA. The pLemiR™ lentiviral vector, which bears a gene for puromycin resistance, was modified by inserting DNA for the pre-microRNA sequence in the 3′ untranslated region of the gene encoding for the TurboRFP red fluorescent protein and driven by the constitutively active cytomegalovirus (CMV) promoter. Following transduction with lentiviruses, and puromycin selection, a stable population of cells named A549/146b was generated. The A549/vec cell-line was generated using pLemiR™ vector without an insert. A549/v146b cells were generated using the vector with a variant human *pre-miR-146b* sequence. In the variant, the nucleic acid segments corresponding to the *miR-146b-5p* and -*3p* microRNA sequences were swapped (figure 1A) with the idea that the variation might result in a relatively higher expression of *miR-146b-3p*, since, among human microRNAs, those generated from the 5p arms of pre-microRNAs tend to be expressed more than those from the 3p arms [11]. The mfold [23] RNA-folding algorithm predicts that like natural *pre-miR-146b*, the variant pre-microRNA too has a stem-loop form (figure 1A), with a ΔG of −31.9 kcal/mol which is 5.2 kcal/mol more than that of the natural pre-microRNA.

Strong and stable expression of the pre-microRNA-bearing TurboRFP gene in >90% of cells of each cell-line was confirmed by fluorescence flow cytometry (figure 1B and data not shown). Multiple cellular RNA preparations were assayed for *miR-146b-5p* and *-3p* mature microRNAs by RT-PCR. Overexpression of the *miR-146b* microRNAs was observed in both A549/146b and A549/v146b cell-lines (figure 1C). Compared to A549/vec, *miR-146b-5p* and *-3p* expression, normalized to that of the 45 base-long, housekeeping small nucleolar RNA gene *RNU6B*, were respectively an average of 4.9- and 6.6-fold higher in A549/146b cells. In A549/v146b, they were respectively an average of 2.6- and 6.5-fold higher, suggesting the variant pre-microRNA expressed by it was processed into mature *miR-146b-5p* and *-3p* microRNAs. To more accurately quantify the effect of the variation on the ratio of amounts of *miR-146b-5p* and *-3p* in the cells, synthetic *miR-146b-5p* and *-3p* RNAs were used to generate standard curves to examine the relationship between RT-PCR measurements (C*_q_* values) and RNA amounts. Though assays for both microRNAs had similar RT-PCR kinetics, with each log_2_ unit change in RNA input producing approximately a unit change in the C*_q_* value over a dilution range of 12 log_2_ units, the sensitivity of the *miR-146b-5p* assay was higher by approximately 3.6 C*_q_* units (figure 1D). Therefore, *miR-146b-3p* C*_q_* values were adjusted by subtracting 3.6 in order to compare the amounts of *miR-146b-5p* and *-3p* RNAs. In all the three A549-derived cell-lines, *miR-146b-5p* microRNA was present at an amount that was about 4-6-fold higher than that of the *-3p* microRNA (figure 1E). The variation in the pre-microRNA sequence was found to have no significant effect on the ratio of amounts of *miR-146b-5p* and *-3p* in the A549/v146b cells compared to the A549/146b cells. Examination of RNA from the human 293T embryonic kidney, BEAS-2B normal bronchial, MCF-7 breast cancer, and ML-2 acute myeloid leukemia cells, indicated that in these cell-lines too, *miR-146b-5p* is present at a 4–22-fold higher molar excess than *-3*p (figure 1E). Thus, the mature microRNA generated from the 5p strand of *pre-miR-146b* appears to be much more prevalent than the one from the 3p strand, as suggested by other studies [8], [9]. Expression of miR-146b-5p in 293T, BEAS-2B, MCF-7 and ML-2 cells was 23.8-, 0.7-, 0.6- and 2.8-fold of that in A549/vec.

### Overexpression of *miR-146b* microRNAs has only a minor effect on the A549 cell phenotype

**Figure 2:**
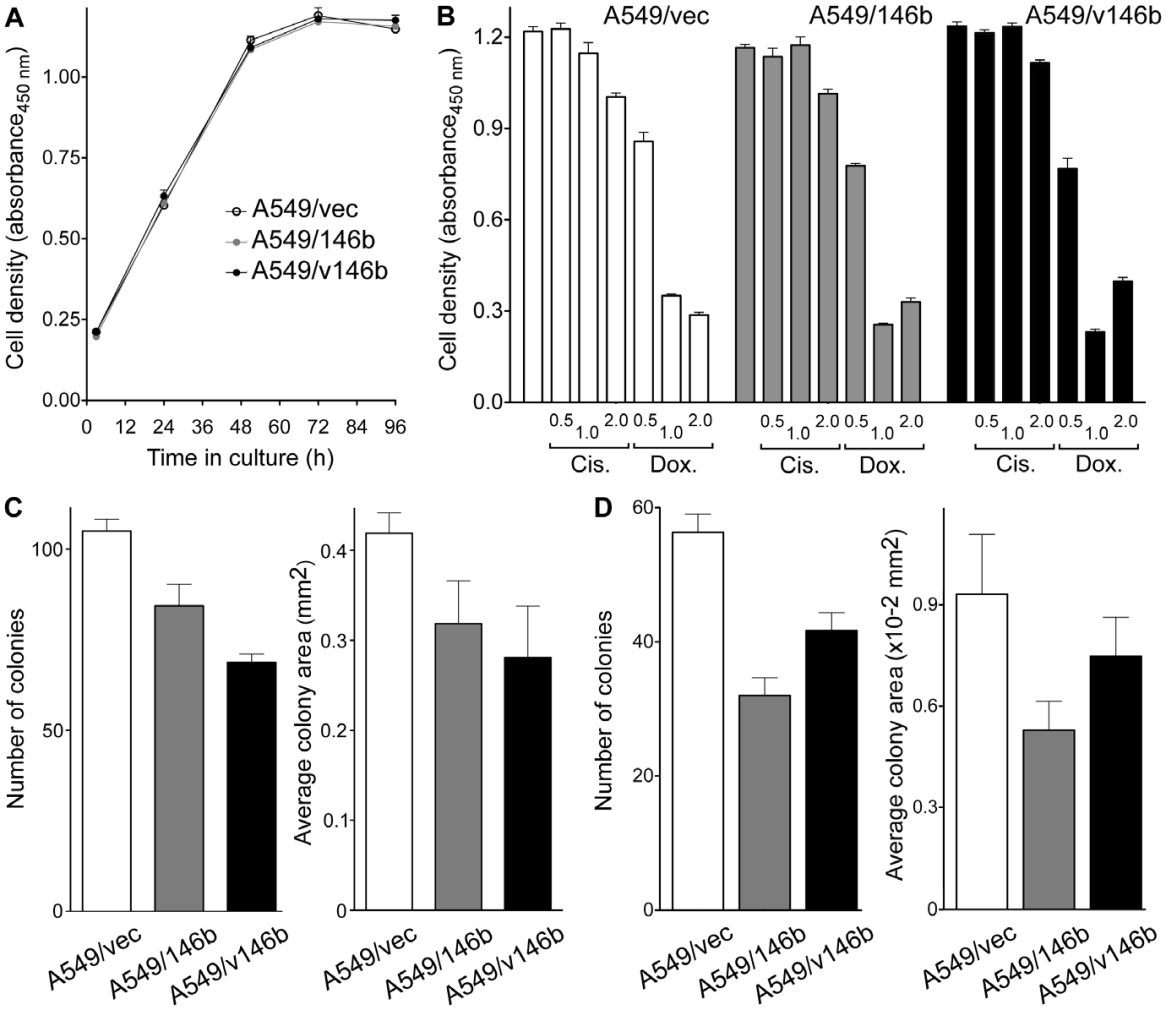
Proliferation of the A549-derived cell-lines. *A*. Cell density was indirectly assessed by a colorimetric assay for cell viability. Absorbance values at 450 nm with increasing hours of cell culture are shown. *B*. Effect on cell density following treatment with 0.5, 1.0 or 2.0 ug/ml cisplatin (*Cis.*) or doxorubicin (*Dox.*) for 2 days. Absorbance values at 450 nm obtained with a colorimetric cell viability assay are shown. *C*, *D*. Number and average areas of colonies seen in adherent (*C*) or non-adherent (*D*) culture on tissue culture plates or in soft agar, respectively. Two hundred (*C*) or 150 (*D*) cells were seeded per culture and colonies were quantified after two (*C*) or six (*D*) weeks. Means and their standard errors for triplicates (*A*, *C*, *D*) or quintuplicates (*B*), from one of three experiments with similar results for *A*, *C* and *D*, are shown.

The A549-derived cell-lines were examined to identify an effect of *miR-146b* overexpression on the A549 cells. Cell viability measurements over time suggested that, compared to A549-vec, both A549/146b and A549/v146b proliferated similarly in cell-culture (figure 2A). Increased *miR-146b* levels did not seem to affect the sensitivity of the cells to either cisplatin or doxorubicin at drug concentrations varying from 0.5 to 2 µg/ml, even though the drugs were clearly cytotoxic to the cells at the highest concentration (figure 2B). Both are anti-cancer drugs that are used for treating NSCLC. However, with the overexpression of the microRNA, both A549/146b and A549/v146b cells formed only 80% and 65%, respectively, of as many colonies as A549/vec did from individual cells in adherent cell culture on the plastic surface of tissue culture plates (t test P values of 0.04 and <0.01, respectively). As expected from the result of the proliferation assay showed in figure 2A, the colonies formed by A549/146b and A549/v146b were not significantly different in size than the ones formed by A549/vec (t test P values of >0.05; figure 2C). Figure S1A shows a similar result obtained in a replicate experiment. Similarly, in non-adherent cell cultures in soft agar, the A549/146b and A549/v146b formed significantly less colonies than A549/vec (57% and 74%, with t test P values of 0.02 and <0.01, respectively), but the sizes colonies were of similar sizes (t test P values of >0.05; figure 2D). There was no significant difference between the colony formation abilities of A549/146b and A549/v146b. Figure S1B shows a similar result obtained in a replicate experiment.

**Figure 3 pone-0022379-g003:**
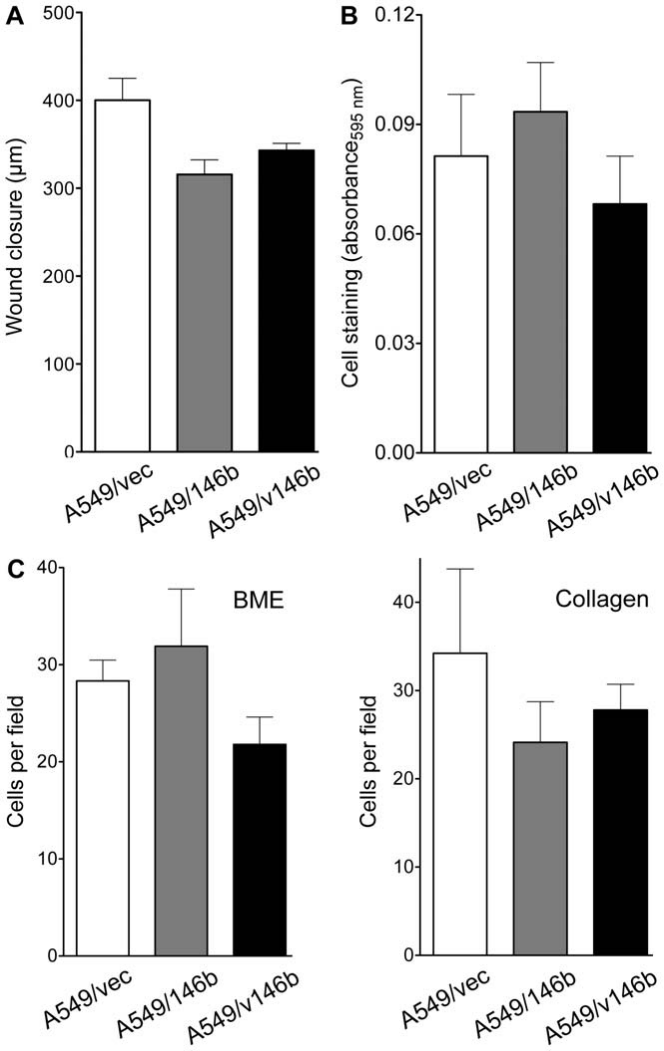
Migration and invasion assays of the A549-derived cell-lines. *A*. Migration of cells in an in vitro wound-healing assay is depicted as a reduction in the width of a scratch across a monolayer of cells after a day of culture. *B*. Migration of serum-starved cells towards 15% serum-containing medium through an 8 µm pore-sized polycarbonate membrane after 8 hours of culture. Migrated cells were fixed and stained with crystal violet, and indirectly quantified by measuring absorbance of extracts of cell stains at 595 nm. *C*. Like *B*, but with membranes coated with a murine basement membrane extract (*BME*) or rat collagen I, and culture for 16 hours. Migrated cells were imaged by red fluorescence microscopy at 5x magnification. Means and their standard errors for triplicates from one of three experiments with similar results are shown.

**Figure 4:**
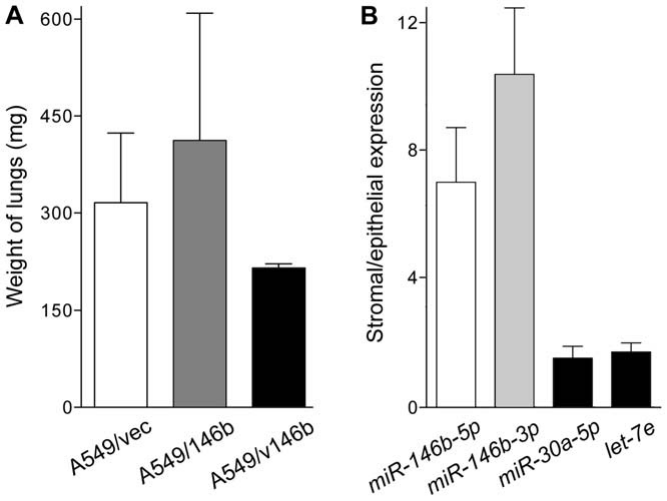
Lung colony formation by the A549-derived cell-lines, and *miR-146b* expressions in tumor stroma and epithelia. *A*. Generation of pulmonary tumors by intravenous injection of the A549-derived cell-lines. A million cells per animal were injected in the tail veins of young female immunodeficient mice. After 12 weeks, animals were dissected and both of their lungs weighed. Five mice were used for each cell-line. *B*. Relative expression of *let-7e*, *miR-30a-5p*, and *miR-146b-5p* and *-3p* microRNAs in stromal and cancerous epithelial components of stage I non-small cell lung cancer as assessed using reverse transcription followed by PCR. Stroma and epithelia were microdissected using tumor sections from 12 cases of the disease. Levels are normalized to that of the *RNU6B* small nucleolar RNA. Means and their standard errors are shown. MicroRNA *miR-146b-3p* was detectable in both compartments of only 11 tumors. Only four tumors were used for *let-7e* quantification.

Migration of the cells was assessed in two different types of assays: an in vitro wound healing assay to test the ability of a cell monolayer to close a gap created by a scratch, and a transwell assay to test the movement of serum-starved cells towards serum-rich medium. In neither assay was the ability of A549/146b or A549/v146b cells significantly different from that of A549/vec, or each other, indicating a lack of an effect of *miR-146b* overexpression on migration of A549 cells (t test P values of >0.05; figure 3A and 3B). Figures S1C and S1D show similar results obtained in replicate experiments. Similar observations were obtained from invasion assays which examined the capability of serum-starved cells to move to serum-rich medium by invading through a layer of extracellular matrix made of either rat collagen I or murine basement membrane extract (figure 3C). Figure S1E and S1F show similar results obtained in replicate experiments. The ability of the cells to migrate and invade was also appraised in one experiment, in an in vivo setting in which the cells were intravenously injected in immunodeficient female mice (five animals for each cell-line), and tumor formation by the cells in the lungs of the mice was examined after 12 weeks. Though none of the mice had exhibited any symptom suggestive of pulmonary tumor formation, such as labored breathing, the period of 12 weeks was chosen based on other studies that used a method similar to the one used here and reported presence of gross pulmonary tumors after injection of A549 cells [24]. Multiple tumors of >1 mm diameter were visible to the naked eye on the outer surface of the lungs in 11 of the 15 mice. Red fluorescence of the tumors indicated that they were derived from the injected cells. However, a significant difference between the lung tumor formation abilities of three lines could not be discerned by such visual examination, or by weighing the lungs (t test P values of >0.05; figure 4A). No tumors on the surface of other thoracic or abdominal organs, or in the linings of the thoracic or abdominal cavities were visible to the naked eye in any of the mice.

### *miR-146b* overexpression altered the expression of only a few genes in A549/146b cells

A549/vec and A549/146b cells of similar age in culture were concurrently grown to semi-confluence on two different occasions for isolating total RNA. A high-density bead-array platform (Illumina® BeadChips™) was used to quantify about 25,000 mRNA transcripts in the RNA preparations to obtain two pairs of gene expression profiles. Forty-three% and 38% of the transcripts were considered as expressed, respectively, in at least one or all four profiles. Pearson correlation coefficients for all expression values for the two biological duplicates for each cell-line were >0.99. Paired comparison of the expression values using moderated t statistics and Benjamini-Hochberg correction for false discovery showed that only 33 transcripts, about 0.3% of expressed mRNA transcripts, were differentially expressed in A549/146b, with 16 down-regulated 30%–50% and 17 up-regulated 50%–140% (table S1). The March 2011 release of the comprehensive miRWalk database of validated and predicted microRNA targets [25] was searched to find out if any of the differentially expressed transcripts was a predicted target of *miR-146b-5p* or *-3p* (table S1). Nine (56%) of the 14 down-regulated known mRNAs were predicted to be a target of at least one of the two microRNAs as per at least one of the following nine microRNA target prediction algorithms: Diana-microT, miRanda, miRDB, miRWalk, PICTAR5, PITA, RNA22, RNAhybrid and TargetScan [25]. It should be noted that 12 (71%) of the 17 up-regulated mRNAs were also predicted to be a *miR-146b* target.

### Expression of *miR-146b* microRNAs is higher in stroma than in epithelia in stage I NSCLC

In the studies in which the *miR-146b* microRNAs in early stage NSCLC were quantified [3], [4], the analyzed RNA was obtained from whole tumor. Besides cancerous epithelial cells, tumors have stromal cells which are not cancerous but which do express microRNAs, and which have an influence on the biological behavior of the cancerous cells of the tumors. To characterize the expression of *miR-146b* in the stromal and epithelial components of NSCLC, a section each from 12 stage I tumors was used for microdissecting and separating the components with LCM. Figure S2 illustrates the microdissection of tumor epithelia and stroma for two of the 12 tumors. Eight of the tumors were squamous cell carcinomas, three were adenocarcinomas, and one was bronchioloalveolar carcinoma. MicroRNA expression values in the two components were compared after normalization to that of *RNU6B* (figure 4B). In all 12 tumors, expression of *miR-146b-5p* was significantly higher in the stromum than in the epithelium (average and range of 7.0- and 1.8-24.1-fold; t test P<0.01). In the 11 tumors in which *miR-146b-3p* was detected in both compartments, expression of *miR-146b-3p* too was higher in the stromum than in the epithelium (average and range of 10.4- and 0.4–25.1-fold, respectively; t test P<0.01). Such relatively high stromal/epithelial expression ratios were not observed for *miR-30a-5p* (average and range of 1.5- and 0.1–4.8-fold, respectively; t test P = 0.95) and *let-7e* (average and range of 1.7- and 1.0–2.2-fold, respectively; t test P = 0.18), two other microRNAs known to be expressed in NSCLC [3].

### Prognostic value of *miR-146b* expression in stage I NSCLC

**Figure 5:**
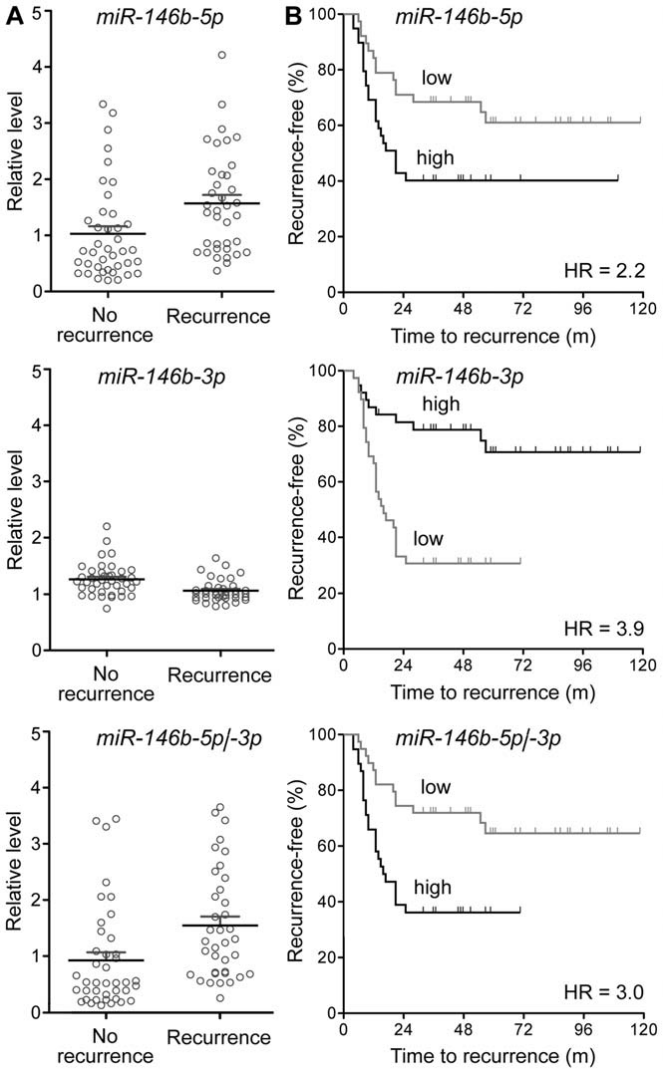
Expression of *miR-146b-5p* and *-3p* in a set of 77 cases of stage I non-small cell lung cancer. *A*. Scatter-plots of microarray signal intensities, relative to that for a reference RNA, for *miR-146b-5p* or *-3p*, or their ratio, for RNA from resected tumor tissue from 37 cases with recurrence of the disease, and for 40 cases without recurrence during the ≥32 months of follow-up following surgery [3]. Means and their standard errors are shown. *B*. Kaplan-Meier plots depicting post-resection recurrence-free survival among the case-groups with values of *miR-146b-5p* or *-3p*, or their ratio, lower (*low*, grey line) or higher (*high*) than the group median. Hazard ratios (*HR*) and time in months to recurrence following surgery are shown. Tick-marks along the lines indicate right-censored data-points.

We analyzed microarray-based quantifications of *miR-146b-5p* and *-3p* in a microRNA expression dataset previously obtained by us for RNA from formalin-fixed, paraffin-embedded tumor tissue from 77 cases of stage IA NSCLC. Thirty-seven (48%) of the cases had a recurrence of the disease following resective surgery while the remaining 40 did not during the minimum of 32 months for which they were followed. MicroRNA expression profiles of their NSCLC tissues were obtained to test the association of recurrence with microRNA alterations, and to generate microRNA classifiers capable of predicting recurrence [3]. MicroRNA *miR-146b-5p* was identified as a component of the multi-variable predictive classifiers in that study. We now specifically compared the expression of *miR-146b-5p* and -*3p* microRNAs, and their ratio, in patients with recurrence to those without. Using the median values of the three variables as cut-off, we also performed survival analyses. As shown in figure 5A, the expression level of *miR-146b-5p* was significantly higher (average 1.53-fold; t test P<0.01) among the cases with recurrence than in those without. However, the expression level of *miR-146b-3p* was significantly lower (average 0.84-fold; t test P<0.01). The ratio of *miR-146b-5p* and *-3p* expression levels was also significantly different between the two case-groups, it being an average of 1.67-fold higher in cases with recurrence (t test P <0.01). Kaplan-Meier survival analyses using group medians for cut-off showed that all three parameters had prognostic value for predicting recurrence of the disease following surgery. High *miR-146b-5p* expression, low *miR-146b-3p* expression, and high *miR-146b-5p*/*-3p* ratio were associated with hazard ratios of 2.23 (95% confidence interval [CI]  = 1.14–4.34), 3.91 (95% CI = 1.99–7.68), and 2.95 (95% CI = 1.50–5.82), with Mantel-Cox log-rank test P values of 0.02, <0.01, and <0.01, respectively (figure 5B). Thus, the two *miR-146b* microRNAs were found to have opposite prognostic values.

## Discussion

The expression of *miR-146b* microRNAs in NSCLC has been associated with prognosis of the disease [3], [4]. We attempted to explore the biological bases of this association by studying the functional consequences of *miR-146b* overexpression on the malignant nature of the A549 lung adenocarcinoma cell-line. Because tumors in NSCLC with a poor prognosis have higher *miR-146b-5p* expression [3], [4], A549 cells overexpressing *miR-146b* microRNAs were expected to have a more malignant behavior. However, an opposite, though minimal, effect of *miR-146b* overexpression on A549 cells was observed. Though the two different A549 overexpressor cell-lines used in this study, A549/146b and A549/v146b, had similar cellular proliferation, chemosensitivity to cisplatin or doxorubicin, and migration and invasiveness both in vivo and in vitro as the A549/vec control cells (figures 2A-[Fig pone-0022379-g003] 4A), they formed about a quarter less colonies in adherent as well as non-adherent cell culture (figures 2C and 2D). Such inhibitory effect of *miR-146b* microRNAs on a cell's malignant phenotype has been demonstrated for breast cancer and glioblastoma cells in studies that found dramatic effects of *miR-146b* overexpression or inhibition on cell migration and invasiveness [18], [19], [20], [21]. The absence of an effect on cell migration and invasiveness in this study (figures 3A-4A) could be because of differences between the cell-lines used in the studies. Among lung cancer cell-lines, A549 cells are considered as highly invasive [26], and it is possible that an effect of *miR-146b* overexpression on cell migration and invasiveness could not be observed because of this. The effects on cell migration and invasiveness seen in the other studies could also be because of the higher degree of *miR-146b-5p* overexpression that was achieved in the other studies. The ∼2.6-6.6-fold overexpression of the *miR-146b* microRNAs in this study is, however, similar to or higher than the general degree of overexpression seen when comparing microRNA profiles of NSCLC tumors with different prognoses [3], [4] (figure 5A).

Examination of gene expression profiles of the A549/vec and A549/146b cells showed that only about 0.3% of the cells' transcriptome was affected by the overexpression of the *miR-146b* microRNAs (table S1). This small fraction appears to be consistent with the observation that A549/vec and A549/146b cells behaved similarly in almost all the assays for cell phenotype that were conducted in this study. We were unable to fathom a connection between the gene expression changes and the reduced ability of A549/146b cells to form colonies in vitro (figures 2C and 2D). More than half of the down-regulated transcripts were predicted to be *miR-146b* targets (table S1), which is consistent with the fact that microRNAs induce target mRNA degradation [27]. A majority of the up-regulated transcripts, too, were predicted to be *miR-146b* targets (table S1). It is possible that the increased levels of these transcripts are a distant effect of *miR-146*b overexpression or reflect different genomic changes in the A549/vec and A549/146b cells during generation of the engineered cell-lines. Many of the up-regulated transcripts are associated with cancer biology; e.g., *ADAM19* [28] and CDH2 [29]. It is interesting that the products of many of the up-regulated transcripts, like *ABCA1*, *GOLT1B* and *SLC30A7*, participate in cellular trafficking of molecules.

The negative association of *miR-146b-5p* levels and malignant nature of cells seen in this and the other cell-line-based studies appears to contradict the observation of higher *miR-146b-5p* expression in tumors with poor prognosis [3], [4], [5]. In the cell-line studies, *miR-146b-5p* overexpression was achieved using expression vectors for *pre-miR-146b*, which is also the precursor for *miR-146b-3p*. Because of this, the level of *miR-146b-3p* too gets higher in the overexpressor cells (figure 1C), and this can affect the influence of *miR-146b-5p* overexpression on the cells. As depicted in figure 5, the prognostic values associated with *miR-146b-5p* and *-3p* microRNAs appear to be antagonistic, with higher *miR-146b-5p* levels but lower *miR-146b-3p* levels in stage I NSCLC tumors associated with disease recurrence following surgical resection. It is thus possible that two microRNAs have opposing effects on the malignant phenotype of cancerous cells, and the effects seen in the cell-line-based studies reflect the net effect of having higher levels of both of them. As per the MicroCosm database of predicted targets of microRNAs (http://www.ebi.ac.uk/enright-srv/microcosm) generated using themiRanda 3.0 algorithm [30], 941 and 943 human transcripts, respectively, are targeted by *miR-146b-5p* and *-3p*, with only 75 targeted by both. Finally, as has been seen in many studies [31], [32], [33], it is possible that the prognostic values of the two microRNAs actually resides within microRNA expression changes in the non-cancerous stroma of the NSCLC tumors. Both *miR-146b-5p* and *-3p* microRNAs appear to be expressed much more in the stroma than in the cancerous epithelia of NSCLC tumors (figure 4B). It should be noted that the *miR-146b* microRNAs are expressed well in cells of the hematopoietic system [34], [35], [36]. A549 is an adenocarcinoma cell-line whereas in the studies identifying the prognostic value of *miR-146b* microRNAs in NSCLC, the tumors were either only squamous cell carcinomas [4] or included squamous cell carcinomas and bronchioloalveolar carcinomas besides adenocarcinomas [3]. It might be that the absence of a major effect of *miR-146b* overexpression on A549 cells reflects the possibility that *miR-146b* expression does not have a prognostic value in NSCLC of adenocarcinoma histology.

MicroRNA overexpression in cells is routinely performed using pre-microRNAs which are produced endogenously from expression constructs or introduced exogenously synthesized and introduced in cells using techniques like RNA transfection. However, because such precursor RNAs give rise to not one but two mature microRNAs from the two strands of the duplex RNA stem of the molecules, as seen in this study, the interpretation of results can be difficult. Engineering the expression of only one of the two microRNAs, or the overexpression of one relative to the other, using precursor RNAs has not been described. Among human microRNAs, 5p microRNAs tend to be expressed more than 3p ones [11], and we sought to achieve relative overexpression of *miR-146b-3p* by using a variant *pre-miR-146b* pre-microRNA in which the segments of the molecule for the 5p and 3p microRNAs were swapped (figure 1A). However, this strategy was unsuccessful (figure 1E). Specific functional overexpression of either the 5p or the 3p microRNA may be possible using pre-microRNAs that have mutations in the sequence of one of the two microRNAs, or at positions that affect pre-microRNA processing [37]. Strand-specific 5′ *O*-methylation or biaryl modification of microRNA duplexes may also be used for 5p- or 3p-specific overexpression [38], [39]. Specific knockdown of a 5p microRNA without affecting its 3p sibling may be achievable using antisense oligonucleotides [40] or microRNA sponges [41]. However, there are different issues such as antisense effects on pre-microRNA processing, non-specificity, and durability of knockdown that are associated with these methods.

In conclusion, we found that overexpression of the lung cancer prognostic *miR-146b* microRNAs diminishes the malignant phenotype of the A549 lung cancer cells. This effect was minimal and opposite of that expected from tumor microRNA expression profiles. The study highlights the myriad issues that impede the elucidation of biological roles of microRNAs and their correlations with observations obtained in tumor microRNA expression studies.

## Supporting Information

Figure S1 **Results of replicate experiments.** Results of one replicate experiment each for the sets of experiments whose results are presented in figures 2C (*A*, adherent colony formation), 2D (*B*, colony formation in soft agar), 3A (*C*, in vitro wound-healing), 3B (*D*, Transwell™ migration), 3C (*E*, invasion through murine basement membrane extract) and 3D (*F*, invasion through rat collagen I). The details provided in the legends of those figures are true for the ones here as well.
(TIF)

Figure S2 **Microdissection of tumor stroma and epithelia.** Photomicrographs of hematoxylin-eosin-stained sections of two non-small cell lung carcinoma tumors (*1* and *2*) before laser microdissection (*A*), after microdissection of tumor epithelia (*B*) and after additional microdissection of tumor stroma adjacent to the dissected epithelia (*C*). Both tumors are squamous cell carcinomas. Tumor epithelia and stroma were identified by histology. Red and blue outlines seen on the images show the enclosed tissue areas that were dissected. Each bar in the scale below is indicates 400 um.
(TIF)

Table S1 **mRNA transcripts differentially expressed in A549/vec and A549/146b cells.**
(DOC)
