# Supplementary figures and images for: Overexpression of the Lung Cancer-Prognostic miR-146b MicroRNAs Has a Minimal and Negative Effect on the Malignant Phenotype of A549 Lung Cancer Cells
Source: PLoS One. 2011 Jul 18;6(7):e22379. doi: 10.1371/journal.pone.0022379 (PMC3138784; doi:10.1371/journal.pone.0022379)

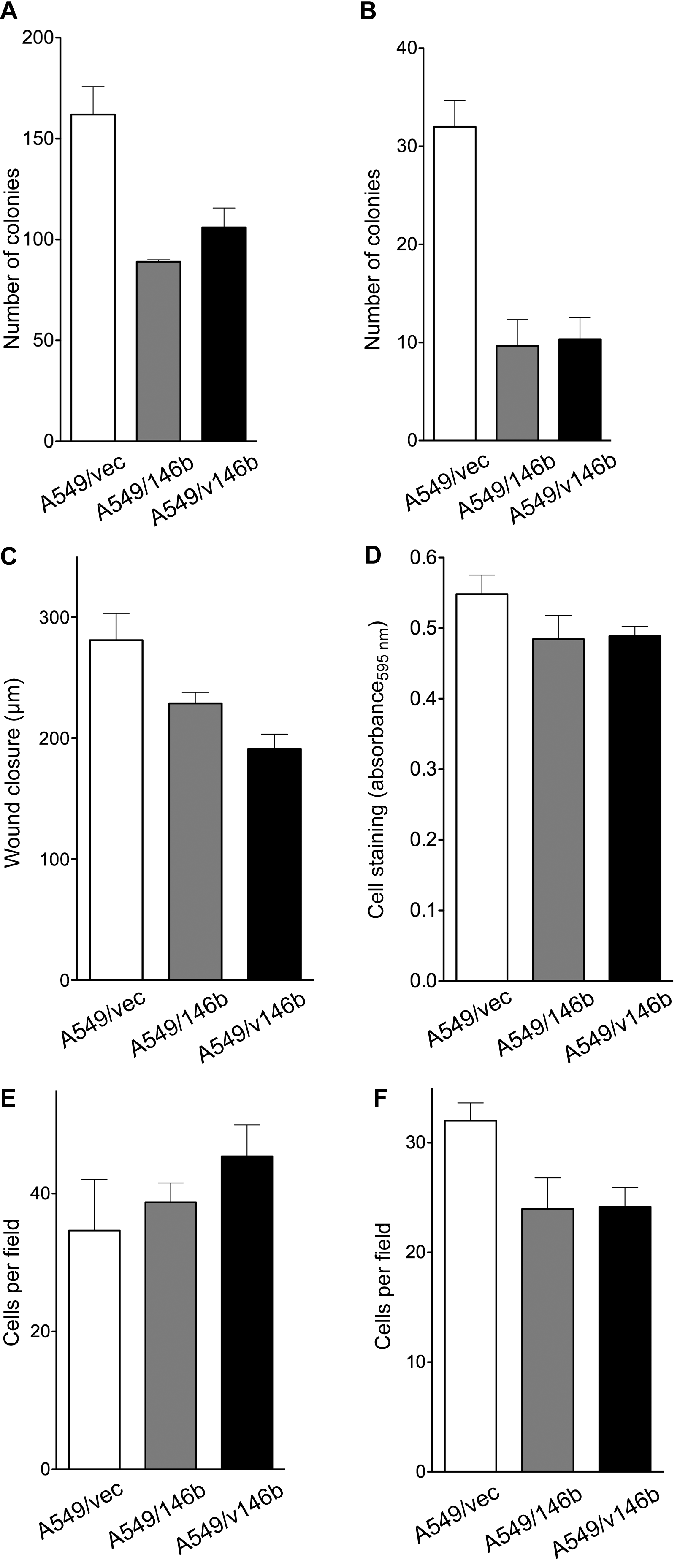

Supplement: Figure S1 — Results of replicate experiments. Results of one replicate experiment each for the sets of experiments whose results are presented in figures 2C (A, adherent colony formation), 2D (B, colony formation in soft agar), 3A (C, in vitro wound-healing), 3B (D, Transwell™ migration), 3C (E, invasion through murine basement membrane extract) and 3D (F, invasion through rat collagen I). The details provided in the legends of those figures are true for the ones here as well. (TIF) [file pone.0022379.s001.tif]

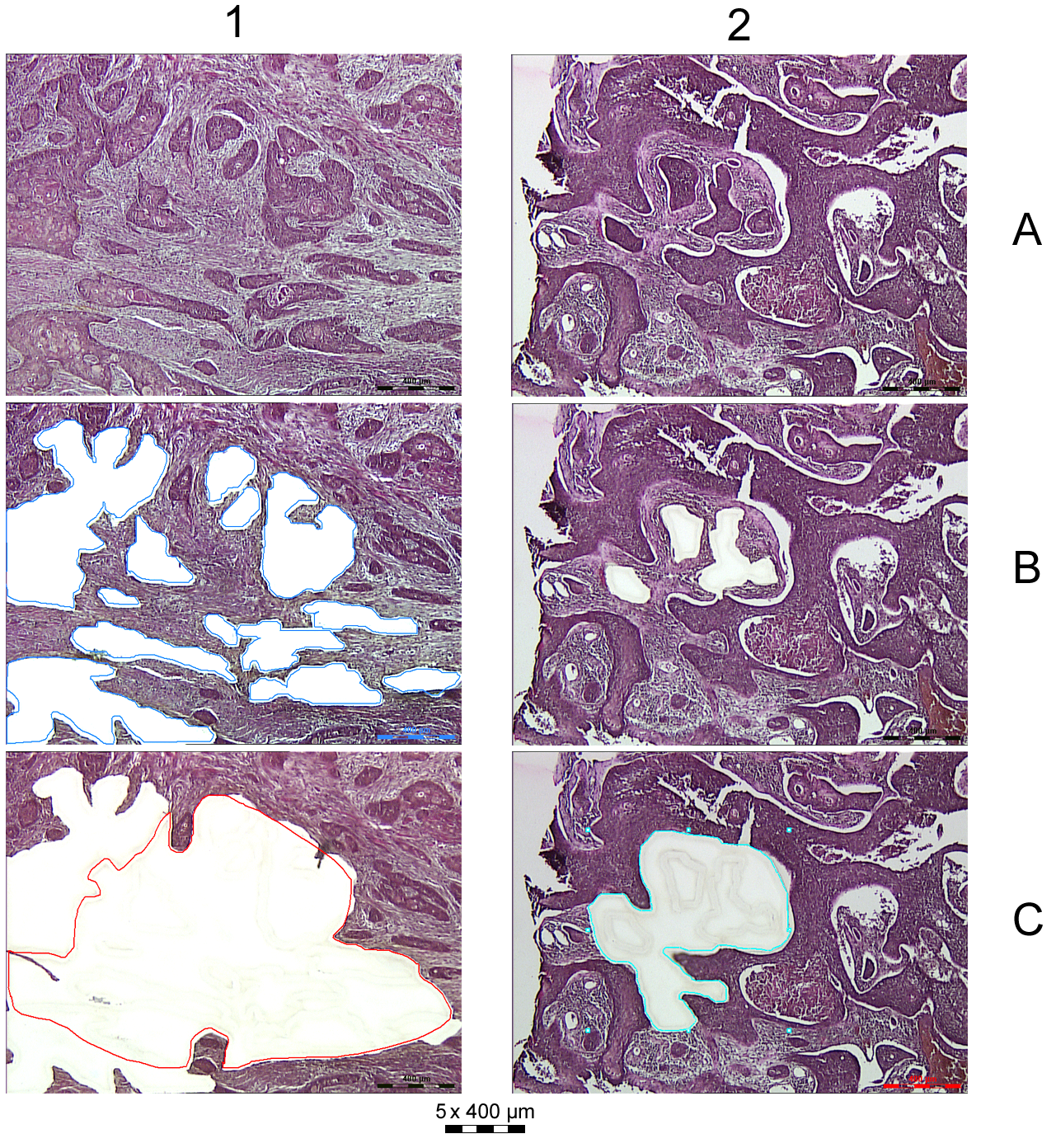

Supplement: Figure S2 — Microdissection of tumor stroma and epithelia. Photomicrographs of hematoxylin-eosin-stained sections of two non-small cell lung carcinoma tumors (1 and 2) before laser microdissection (A), after microdissection of tumor epithelia (B) and after additional microdissection of tumor stroma adjacent to the dissected epithelia (C). Both tumors are squamous cell carcinomas. Tumor epithelia and stroma were identified by histology. Red and blue outlines seen on the images show the enclosed tissue areas that were dissected. Each bar in the scale below is indicates 400 um. (TIF) [file pone.0022379.s002.tif]
