# Supplementary material for: Overexpression of the Lung Cancer-Prognostic miR-146b MicroRNAs Has a Minimal and Negative Effect on the Malignant Phenotype of A549 Lung Cancer Cells
Source: PLoS One. 2011 Jul 18;6(7):e22379. doi: 10.1371/journal.pone.0022379 (PMC3138784; doi:10.1371/journal.pone.0022379)
Supplement: Table S1 — mRNA transcripts differentially expressed in A549/vec and A549/146b cells. (DOC) [file pone.0022379.s003.doc]

**Table S1**

mRNA transcripts differentially expressed in A549/vec and A549/146b cells

| *Name* | *Symbol* | *Fold-change*^1^ | *P value*^2^ | *miR-146b target prediction^3^* |
| --- | --- | --- | --- | --- |
| ADAM metallopeptidase domain 19 (meltrin beta) | *ADAM19* | 1.6 | 0.0112 | 5p: Diana-microT, miRanda, miRWalk, PITA, TargetScan; 3p: 5p: miRanda, miRWalk, PITA, TargetScan |
| Adaptor-related protein complex 1, gamma 2 subunit | *AP1G2* | 0.7 | 0.0431 | (not a target) |
| Annexin A10 | *ANXA10* | 1.8 | 0.0005 | (not a target) |
| ATP-binding cassette, sub-family A (ABC1), member 1 | *ABCA1* | 1.6 | 0.0081 | 5p: PITA; 3p: miRanda, miRWalk, PITA, TargetScan |
| Cadherin 2, type 1, N-cadherin (neuronal) | *CDH2* | 1.7 | 0.0014 | 5p: PITA |
| Carbamoyl-phosphate synthetase 1, mitochondrial | *CPS1* | 1.6 | 0.0106 | 3p: miRanda, miRDB, PITA |
| Cathepsin L1 | *CTSL1* | 0.6 | 0.0085 | (not a target) |
| CDK5 regulatory subunit associated protein 3 | *CDK5RAP3* | 0.6 | 0.0034 | 5p: miRanda, PITA; 3p: miRanda, PITA |
| CDK5 regulatory subunit associated protein 3 | *CDK5RAP3* | 0.7 | 0.0498 | 5p: miRanda, PITA; 3p: miRanda, PITA |
| Claudin 15 | *CLDN15* | 0.7 | 0.0498 | (not a target) |
| Collagen, type IV, alpha 1 | *COL4A1* | 1.5 | 0.0475 | 5p: PITA; 3p: miRanda, PITA |
| FBJ murine osteosarcoma viral oncogene homolog | *FOS* | 0.7 | 0.0378 | 5p: PITA |
| FBJ murine osteosarcoma viral oncogene homolog B | *FOSB* | 0.6 | 0.0025 | 5p: miRanda, PITA; 3p: miRanda, PITA |
| Inhibitor of DNA binding 1, dominant negative helix-loop-helix protein | *ID1* | 0.6 | 0.0146 | 5p: PITA |
| Maternally expressed 3 (non-protein coding) | *MEG3* | 0.5 | <0.0001 | (not a target) |
| Paired immunoglobin-like type 2 receptor beta | *PILRB* | 0.7 | 0.0378 | (not a target) |
| Prickle homolog 2 (Drosophila) | *PRICKLE2* | 1.5 | 0.0299 | 3p: miRanda |
| Protein phosphatase 1, catalytic subunit, beta isoform | *PPP1CB* | 1.5 | 0.0498 | 5p: Diana-microT, miRanda, miRWalk, PITA, TargetScan; 3p: miRanda, PITA |
| Regulator of G-protein signaling 11 | *RGS11* | 0.7 | 0.0299 | 5p: PITA |
| Ribosomal protein L9 | *RPL9* | 1.6 | 0.0239 | 3p: miRWalk, TargetScan |
| Ribosomal protein S27 | *RPS27* | 1.6 | 0.0296 | (not a target) |
| Ribosomal protein S28 | *RPS28* | 1.5 | 0.0378 | (not a target) |
| Small nuclear ribonucleoprotein 70 kDa (U1) | *SNRNP70* | 0.6 | 0.0239 | (not a target) |
| Golgi transport 1 homolog B (S. cerevisiae) | *GOLT1B* | 1.5 | 0.0299 | (not a target) |
| Solute carrier family 30 (zinc transporter), member 7 | *SLC30A7* | 1.5 | 0.0332 | 5p: PITA; 3p: PITA |
| Transmembrane and tetratricopeptide repeat containing 3 | *TMTC3* | 1.5 | 0.0312 | 5p: PITA |
| Trinucleotide repeat containing 6B | *TNRC6B* | 1.6 | 0.0106 | 5p: PITA; 3p: PITA |
| Tyrosine 3-monooxygenase/tryptophan 5-monooxygenase activation protein, epsilon polypeptide | *YWHAE* | 0.5 | 0.0296 | 5p: miRanda, PITA |
| (Unknown gene) |  | 0.5 | 0.0002 | (not a target) |
| (Unknown gene) |  | 0.6 | 0.0034 | (not a target) |
| Vesicle-associated membrane protein 8 (endobrevin) | *VAMP8* | 2.4 | <0.0001 | (not a target) |
| Zinc finger protein 36, C3H type, homolog (mouse) | *ZFP36* | 0.7 | 0.0498 | 5p: miRanda; 3p: miRanda, PITA |
| Zinc finger, DHHC-type containing 11 | *ZDHHC11* | 0.7 | 0.0299 | 5p: PITA; 3p: miRanda, PITA |

^1^A549/146b vs. A549/vec

^2^Benjamini-Hochberg-corrected

^3^Algorithm(s) predicting targeting of the mRNA by the 5p or 3p forms of *miR-146b*
